# Editorial: Health, science and innovation for the future of food system

**DOI:** 10.3389/fnut.2024.1462890

**Published:** 2024-07-25

**Authors:** Giuseppe Poli, Mélanie Charron, Carlo Agostoni, I. Sam Saguy

**Affiliations:** ^1^Unit of General Pathology and Physiopathology, Department of Clinical and Biological Sciences, San Luigi Hospital, University of Turin, Turin, Italy; ^2^Soremartec Ferrero Group, Alba, Italy; ^3^Pediatric Intermediate Care Unit, IRCCS Ca' Granda, Ospedale Maggiore Policlinico, Milan, Italy; ^4^Department of Clinical Sciences and Community Health, University of Milan, Milan, Italy; ^5^Robert H. Smith Faculty of Agriculture, Food and Environment, The Hebrew University of Jerusalem, Jerusalem, Israel

**Keywords:** dietary protein, food allergy, plant food, food processing, sarcopenia

The International Master Michele Ferrero 9th edition program (2022/23) integrated cutting-edge advances in Food Science, Food Technology (FS&T), Nutrition Science, health, innovation and digital proliferation. The curriculum featured novel pedagogical approaches and new product development, emphasizing healthy aging, innovation, adaptability, and sustainability. This program was a collaboration between the Ferrero Foundation, Soremartec, Turin University, and Catholic University of the Sacred Heart, Milan.

This Research Topic is one of the program outcomes, comprises nine papers derived from three seminars and two virtual webinars featuring sixteen globally renowned experts. It focuses on the complex aspects of food allergies and on protein needs, as well as on other recent breakthroughs in FS&T and healthy aging, furnishing most recent insights to augment scientific understanding and possible applications.

- *Food allergies around the world* (Wong):

This review explores the relationship between economic development, urbanization, and the increasing incidence of allergic conditions, with a specific focus on food allergy in infants and young children. In developed countries, one in three children suffer from at least one allergic disorder, including food allergies, eczema, allergic rhinitis, and asthma. Egg, milk, fish, wheat, peanuts and tree nuts are by far the most represented allergens. Identifying environmental and lifestyle factors associated with the notable difference of asthma incidence between urban and rural areas is crucial for developing effective primary prevention measures, particularly in vulnerable populations.

- *Food allergy issues among consumers: a comprehensive review* (Sansweet et al.):

This minireview highlights the multifaceted impact of food allergies (FA) on consumers, underlining its significant influence on social, psychological, and economic aspects of life. It emphasizes the need for increased awareness and education to improve quality of life (QoL) for individuals affected by (FA). The review advocates for collaboration among all stakeholders, including medical professionals, researchers, advocacy groups, and policymakers, to address these challenges. It underscores the importance of ensuring affordable and accessible care for all, highlighting potential solutions to alleviate the burdens faced by those with FA.

- *The future of cow's milk allergy – milk ladders in IgE-mediated food allergy* (Hicks et al.):

The perspective centers on the future management and treatment of cow's milk allergy (CMA), particularly focusing on the use of milk ladders (i.e., a stepwise progression from extensively heated *to less heated food*) in IgE-mediated food allergy. Some of its highlights include: consideration of various management approaches for CMA, including alternative milk sources. Discussion on the potential of milk ladders in CMA management. And emphasis on the need for ongoing research to enhance treatment options.

- *Dietary proteins: from evolution to engineering* (Daniel):

This review delves into the significance of dietary proteins in human nutrition, tracing their role from evolution to current consumption patterns, and future production prospects.. Main points include the importance of dietary proteins, which contain indispensable amino acids, playing a crucial role in human evolution and proper growth and body maintenance. Evolution of hominins in diverse food ecosystems highlighting the centrality of proteins in human nutrition. Future prospects for protein production delving on sustainability and meeting the increasing food demand are also reviewed. The review stresses the need for new protein sources that not only provide essential amino acids but also serve functional roles in food production, considering human physiology and metabolism from an evolutionary perspective.

- *The nutritional support to prevent sarcopenia in the elderly* (Giacosa et al.):

Sarcopenia is a condition characterized by muscle loss with significant health consequences. This review delves into its epidemiology, pathophysiology, and methods for early detection, with a focus on treatment through physical activity and nutritional support. Specific foods designed for sarcopenia management have shown promising results. Combining these interventions with physical activity enhances muscle protein synthesis and strength. Long-term efficacy and tolerability of these interventions require further investigation. Muscle-targeted nutritional supplementation combined with resistance exercise appears to be an effective strategy in preventing sarcopenia in high-risk elderly populations.

- *A review on nutritional quality of animal and plant-based milk alternatives: a focus on protein* (Karoui and Bouaicha):

The report highlights the growing consumer demand for protein-rich products and the nutritional differences between dairy foods and plant-based milk alternatives. Dairy products are recognized for their high-quality proteins, while plant proteins typically have lower essential amino acid content. This fact underscores the importance of ongoing research in improving the nutritional quality of plant-based milk alternatives, mainly by removing the anti-nutritional factors present in plant-based proteins and enhancing the protein content and amino acid composition of these alternatives, potentially by blending different plant proteins.

- *Possible interactions between selected food processing and medications* (Poli et al.):

This novel review highlights possible interactions between selected food processing technologies and medications by exploring both potential benefits and risks. The review delves into examination of thermal processing technologies' effects on drug absorption and metabolism. Innovative food processing technologies for enhanced bioavailability are described. It calls for multidisciplinary intensified scientific research and comprehensive investigations into possible interactions and their underlying mechanisms that are indispensable for ensuring food and medication safety and efficacy. It points to the integration between food processing and drug production underscoring the need for a unified approach for their future development.

- *The taste for health: the role of taste receptors and their ligands in the complex food/health relationship* (Morini):

The relationship between taste, food choices, and health goes beyond pleasure. G protein-coupled receptors (GPCRs) in the gastrointestinal tract detect sweet, umami, and bitter flavors, playing critical roles in nutrient sensing, hormonal regulation, microbiota balance, immune response, and gut-brain communication. These gut-based taste receptors regulate microbiota composition and immune activity. Their discovery has led to potential drug targets and therapeutic interventions. Integrating scientific advancements will enhance food design, support sustainable food production, and drive progress in functional foods tailored to health needs, fostering a holistic approach to nutrition and wellness.

- *ONE QUALITY concept: a narrative perspective to unravel nutritional challenges, controversies, and the imperative need of transforming our food systems* (Menta et al.):

The ONE QUALITY concept perspective advocates for a transformative approach to global nutrition, emphasizing culturally sensitive, evidence-based guidelines. It underscores the need for healthy and sustainable diets tailored to diverse cultural contexts and the importance of food safety policies that engage all stakeholders. The perspective moves away from demonizing specific dietary components like saturated fats and embraces a comprehensive view of diet, including plant-based foods. It highlights the role of international agencies and regulatory bodies in promoting evidence-based practices and addressing environmental constraints on food systems. The perspective also emphasizes the need for a comprehensive data bank to develop a global, science-based approach.

## Author contributions

GP: Conceptualization, Writing – original draft, Writing – review & editing, Supervision, Validation. MC: Formal analysis, Validation, Writing – review & editing. CA: Validation, Writing – review & editing. IS: Conceptualization, Writing – original draft, Writing – review & editing, Supervision, Validation.

